# Professor Rolf Garms: 1931–2021

**DOI:** 10.1007/s00436-022-07482-y

**Published:** 2022-04-04

**Authors:** Norbert W. Brattig, Robert A. Cheke, John B. Davies, Renke Lühken, Andreas Krüger

**Affiliations:** 1 Bernhard Nocht Institute for Tropical Medicine, Hamburg, Germany; 2 Natural Resources Institute, University of Greenwich at Medway, Chatham Maritime, Kent UK; 3 Liverpool School of Tropical Medicine, Pembroke Place, Liverpool, UK; 4 Bundeswehr Hospital Koblenz, Koblenz, Germany


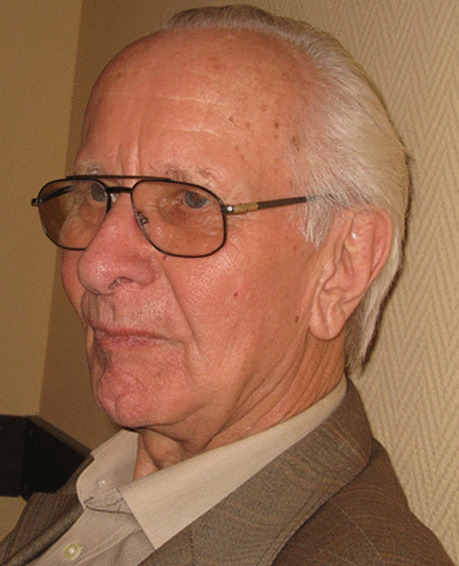


Professor Rolf Garms, 6 October 2011 (Photograph: R.A. Cheke)

Rolf Garms, who died on 5 October 2021, aged 89, was an outstanding and distinguished medical entomologist who began and ended his career at the Bernhard Nocht Institute for Tropical Medicine (BNITM) in Hamburg. Dr. Garms was foremost amongst experts on blackfly biology and on the transmission and control of onchocerciasis.

Rolf Garms began studying zoology at the University of Hamburg in 1950 and was awarded a PhD in 1958. He was employed at the BNITM in 1962 working there until 1996 when he retired, but he continued working as an emeritus professor until his final illness. In 1958 he was appointed Director of the Liberian Research Unit of the BNITM, based at Bong Mine. Rolf Garms achieved his habilitation (an academic procedure common in Germany as a prerequisite to becoming a full professor) in 1984 and the *venia legendi* (an authority to teach, given a successful habilitation) in 1988. In 1992, Dr. Garms was appointed as full professor at the University of Hamburg.

After initiating his career with research on mosquitoes, Rolf began his lifelong fascination with blackflies during extensive and arduous fieldwork in Guinea where he discovered new species, documented the distribution of *Simulium damnosum* s.l., and conducted trials of DDT and fenthion on the latter. He pursued similar research in Liberia and this West African experience led to his subsequent contributions to the World Health Organization’s Onchocerciasis Control Programme in West Africa (OCP). He was involved from the outset, being one of the scientists who attended the first planning meeting in Tunis in 1968 (WHO 1969), which led to the Preparatory Assistance to Governments Mission whose report in November 1973 (WHO 1973) was followed by the foundation of the OCP in January 1974. Dr. Garms was a member of the Scientific and Technical Advisory Committee of the OCP, and he was soon involved as a consultant, especially when the programme could not explain the continued presence of biting flies in controlled zones. Suspecting reinvasions from untreated areas, Rolf masterminded pioneering studies with John Davies and Frank Walsh that demonstrated that savannah members of the *Simulium damnosum* species complex could migrate as far as 500 km. For this work, they were awarded the Düsseldorfer Hygienepreis Silver Medal in 1980. The first reinvasion studies were conducted in the west of the OCP, but, together with Robert Cheke and many other collaborators, Rolf conducted similar studies in the eastern region of the OCP and showed that, in addition to the savannah species, *Simulium squamosum* was also involved in displacements of 150 km or more.

In addition to his reinvasion work, Dr. Garms contributed a great deal of knowledge about the biology and the transmission efficacy of different blackfly taxa, often working in the laboratory and field with his assistant the late Magdalene Kerner. Rolf published 147 articles between 1958 and 2021, i.e. spanning more than 60 years, including analyses of the morphology of *Simulium* species, their cytotaxonomy, phylogeny, ecology, geographic distribution, seasonal variation, transmission abilities, histochemical differentiation, non-*Onchocerca volvulus* and other filarial larvae, anthropophily, and zoophily, mostly in West Africa but also in Guatemala, Uganda, and Yemen. However, apart from his reinvasion studies, Rolf’s tireless and dedicated work in Uganda led to another major achievement: the interruption of the transmission of *Onchocerca* by larvicides in many foci, involving controlling both *S. neavei* group members and *S. damnsoum* s.l.

During his career, Dr. Garms consistently inspired generations of participants on the BNITM courses on tropical infectious diseases with his engaging lectures on parasites and vectors and guided a multitude of doctoral dissertations. Dr. Garms served on the Advisory Committee of the Special Programme for Research and Training in Tropical Diseases (WHO/TDR) and was awarded an honorary membership of the German Society of Parasitology in 2016. He was a fellow of the Royal Society of Tropical Medicine and Hygiene, and for many years, he was one of the editors of the journal *Tropical Medicine and Parasitology* (now *Tropical Medicine & International Health*).

A great deal of Rolf Garms’ research interest was dedicated to the taxonomy of Afrotropical blackflies. He contributed to the description of eight new species. Chronologically these were *Simulium futaense* Garms & Post 1966, *S. geigyi* and *S. weyeri* Garms & Häusermann 1968, *S. liberiense* Garms 1973, *S. audreyae* Garms & Disney 1974, *S. yemenense* Crosskey & Garms 1982, *S. rasyani* Garms, Kerner & Meredith 1988 and *S. pandanophilum* Krüger, Nurmi & Garms 1998. In addition, he was involved in descriptions of the following new cyto- and morphoforms: the ‘Tanzania’ form of *S. mcmahoni* (Garms & Disney, 1974), ‘type III’ and ‘type IV’ forms of *S. sirbanum* (Cheke, Garms, Walsh, & Phillips 1987, confirmed cytotaxonomically by Fiasorgbor & Cheke 1992) and three forms of *S. soubrense*: ‘Beffa’ (Meredith, Cheke, & Garms 1983), ‘Farmington’ and ‘St Paul’ (Güzelhan & Garms 1987). A new freshwater crab, *Potamonautes rukwanzi* Corace, Cumberlidge, & Garms 2001 was also discovered during his fieldwork in Uganda. Furthermore he is remembered in the eponymous *Simulium garmsi* Crosskey 1969, *Onchocerca garmsi* Schulz-Key & Bain 1976 (a parasite of Red Deer *Cervus elaphus*), two Muscid flies *Pyrellina garmsi* Zielke 1971 and *Helina garmsi* Zielke 1974 and the Saturniid moth *Orthogonioptilum garmsi* Bouyer 1995. Also, a pygmy grasshopper (Orthoptera: Tetrigidae) that he collected in Liberia will soon be named after him, reflecting some of his other entomological interests, which also encompassed Tiger beetles (Coleoptera: Cicindelidae).

During his last 10 years, Dr. Garms was involved in research on native European and invasive mosquitoes of metropolitan Hamburg, including studies of host-feeding patterns and the presence of viruses in mosquitoes. Research on native mosquitoes had almost ceased after the disappearance of malaria in Europe. Thus, Rolf concluded his research work on topics with which he had started his scientific career at BNITM in the 1960s, and he continued to support graduate students during sampling campaigns and drafting manuscripts. The demise of Dr. Garms will certainly leave a huge vacuum in the field of transmission and control of onchocerciasis (WHO 1987) and characterisation of the *Simulium* vector. His legacy of hard work and a lifetime’s dedication to his subject will continue to inspire us all, as we remember a humble and charming man, possessed of an enviable intelligence and scientific acumen.

In 1958, Rolf Garms married Elke Biel who survives him, along with their son Gunnar and their daughters Grietje and Janna.
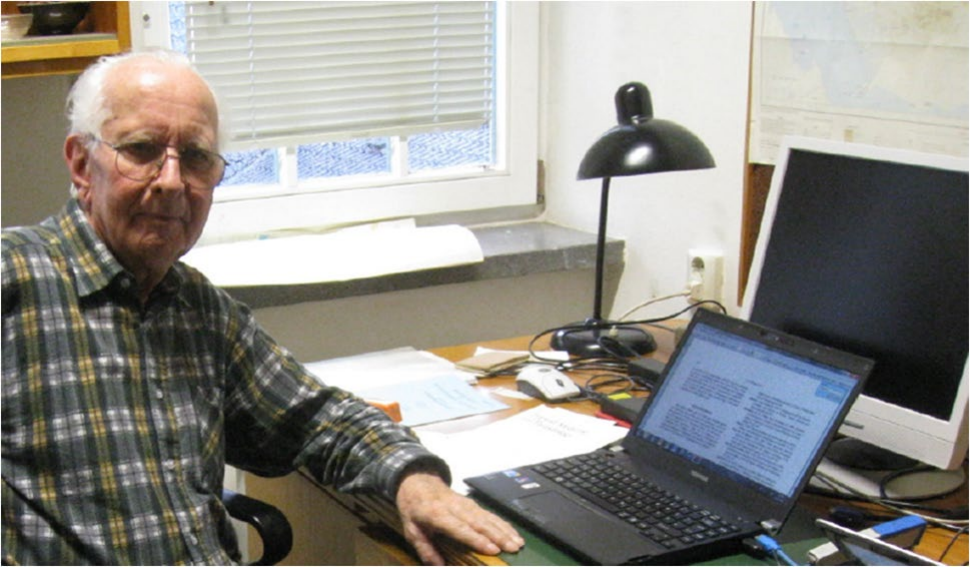


Professor Rolf Garms in his office at the Bernhard Nocht Institute for Tropical Medicine, Hamburg, Germany, 7 October 2015 (photograph: R. A. Cheke)
